# Clinical presentation and management of stable coronary artery disease in Austria

**DOI:** 10.1371/journal.pone.0176257

**Published:** 2017-04-27

**Authors:** Otto Pichlhöfer, Manfred Maier, Roza Badr-Eslam, Robin Ristl, Magdalena Zebrowska, Irene M. Lang

**Affiliations:** 1 Department of General Practice and Family Medicine, Centre for Public Health, Medical University of Vienna, Vienna, Austria; 2 Department of Internal Medicine II, Division of Cardiology, Vienna General Hospital, Medical University of Vienna, Vienna, Austria; 3 Institute of Medical Statistics, Center for Medical Statistics, Informatics, and Intelligent Systems, Medical University of Vienna, Vienna, Austria; University Medical Center, Freiburg, GERMANY

## Abstract

**Background:**

Cardiovascular disease is the main cause of death in Austria. However, no systematic information exists regarding characteristics and treatments of contemporary patients with stable coronary artery disease (CAD) in Austria. We assembled two retrospective physicians’ databases to describe demographics, clinical profiles, and therapeutic strategies in patients with stable CAD. In addition, we compared patient profiles of secondary care internists and hospital-based cardiologists with those of general practitioners in a primary care setting outside of hospital.

**Methods:**

The study population was identified from retrospective chart review of 1020 patients from 106 primary care physicians in Austria (ProCor II registry), and was merged with a previous similar database of 1280 patients under secondary care (ProCor I registry) to yield a total patient number of 2300.

**Results:**

Female patients with stable CAD were older, had more angina and/or heart failure symptoms, and more depression than males. Female gender, type 2 diabetes mellitus, higher CCS class and asthma/COPD were predictors of elevated heart rate, while previous coronary events/revascularization predicted a lower heart rate in multivariate analysis. There were no significant differences with regard to characteristics and management of patients of general practitioners in the primary care setting versus internists in secondary care.

**Conclusions:**

Characteristics and treatments of unselected patients with stable ischemic heart disease in Austria resemble the pattern of large international registries of stable ischemic heart disease, with the exception that diabetes and systemic hypertension were more prevalent.

## Introduction

Coronary artery disease (CAD) has been the major cause of death worldwide. Despite progress in prevention and management of cardiovascular diseases leading to a steady decline of death rates in industrialized countries [1], cardiovascular mortality has increased in low- and middle-income countries because they are adopting a Western lifestyle. Recent data illustrate that the aging and growth of the population has resulted in an increase in global cardiovascular deaths between 1990 and 2013 [2]. Therefore, it is expected that cardiovascular disease will remain the leading cause of death until 2030.

Austria is a good example of a wealthy, industrialized country with easy access to healthcare. In 2011, 437,000 patients in Austria suffered from cardiovascular diseases, corresponding to 5,211 patients per 100,000, or 19% of patients who were admitted to hospitals (http://www.goeg.at/de/GB-Archiv).

In order to understand epidemiology, referral patterns, gender distribution, clinical features and treatment patterns of outpatients with stable CAD in Austria, two retrospective observational cross-sectional registries were established.

ProCor I was based on data collected by Austrian Internal Medicine specialists in 2009 [3]. ProCor I reported excellent contemporary care of patients with stable CAD, yet, lower than expected doses of beta-blockers. ProCor II aimed to analyze and compare data provided by Austrian general practitioners in 2012, assessing patient characteristics, heart rate control, medications and general management practices and quality of patients with stable coronary artery disease under primary and secondary care. In particular, we focused on the association of anginal symptoms and medications with gender and heart rate, two controversial risk factors of stable CAD.

## Methods

### Subjects and methods

The study data were collected as retrospective databases of practicing physicians. Participating internists were approached as described (3); 810 general practitioners (GPs) were approached from the research network of general practitioners of the Department of General Practice and from the list of general practitioners working in the public health care sector holding a contract with all Austrian insurance companies. Inclusion criteria for patients in both studies (Procor I and II) were currently stable CAD based on a history of at least one of the following: 1) Documented myocardial infarction (more than 3 months ago); 2) Coronary angiography showing at least one coronary stenosis of more than 50%; 3) Chest pain with myocardial ischemia proven by stress ECG, stress echocardiography or myocardial nuclear imaging; 4) previous coronary artery bypass graft (CABG) or percutaneous coronary intervention (PCI) (more than 3 months ago). Physicians were asked to record retrospective data of 10 to 15 patients who met inclusion and exclusion criteria.

The questionnaire for ProCor I contained a set of 17 variables, while in ProCor II 24 additional parameters were added. 39 questions were focused on demographics, risk, lifestyle factors, angina pectoris symptoms, measures of heart failure, resting heart rate (HR), and cardiovascular medications. Demographics were age, gender and migrational status. Risk factors and life style parameters were recorded as family history of CAD, hypertension, diabetes, dyslipidemia, peripheral arterial disease (PAD), the general level of physical exercise and smoking status. Responses were categorized as ‘yes’–’no’–’not known’, with the exception of regular exercise, which was categorized as ‘none’–’light’–’intermediate’ (corresponding to one to three times per week) and ‘intensive’ which was corresponding to more than three times per week. Year of CAD diagnosis, and a previous acute coronary syndrome (ACS), myocardial infarction (MI) or percutaneous coronary intervention (PCI), a history of stroke, obstructive respiratory disease (defined as chronic obstructive pulmonary disease (COPD)), and a history of depression were monitored. The questionnaire assessed angina pectoris events (weekly episodes), and the average weekly nitro-glycerine use. The Canadian Cardiovascular Society grading of angina pectoris (CSS) was recorded. Heart failure was rated by New York Heart Association functional classification (NYHA). The presence of atrial fibrillation, previous pacemaker, defibrillator or cardiac resynchronization therapy (CRT) implantation were recorded. Cardiovascular medication was grouped into inhibitors of platelet aggregation—(acetylsalicylic acid (ASS) and others; dual platelet inhibitors such as combinations of ASS with clopidogrel, ticagrelor or prasugrel), oral anticoagulants such as dicoumarol, acenocoumarol, or dabigatran; angiotensin-converting-enzyme (ACE) inhibitors or angiotensin-II-receptor antagonists; lipid-lowering drugs, diuretics, beta-blockers such as atenolol, bisoprolol, metoprolol, nebivolol, carvedilol or others including daily dose; calcium channel blockers (CCB) such as diltiazem, verapamil, amlodipine or others; ivabradine, long lasting nitrates and nicorandil; ranolazin; molsidomin, cardiac glycosides, amiodarone, dronedarone or sotalol.

Approval of the institutional review board of the Medical University of Vienna was obtained (EK Nr: 1162/2011).

### Statistical analysis

All analyses were performed for each of the two study data sets separately, and for the pooled data. Conclusions based on the pooled data were classified as meaningful only under the assumption that both data sets were samples from the same general population. To compare characteristics of ProCor I and ProCor II, T-Tests were calculated for metric variables and Chi-squared tests for categorical variables. The relative frequencies of patients receiving selected medications were depicted by bar-plots. Simple linear regression models were estimated for heart rate explained by each potential predictor separately. For categorical predictors the estimated regression coefficients correspond to the differences in heart rate between the respective class and the reference class. 95% confidence intervals for the regression coefficients were calculated. The null hypothesis of a coefficient being equal to zero was tested by a t-test and the global null hypothesis of no effect of a specific predictor was tested by an F-test.

#### Missing data

The target variable “heart rate” had 12 missing observations in Procor I (0.9%) and 18 missing observations in Procor II (1.8%). In ProCor I 8% of variables were missing, and 12% in ProCor II. In no patient were many missing observations. Models for heart rate including individual predictors were calculated based on available data for a given variable, assuming that data were missing at random. For multiple regression models this approach was not feasible, because ideal sets of observations were available for too few patients. Therefore, multiple imputation method by chained equations was used to complete the data. The “R” package “mice” was used [4] where the imputation method is established. In the present work 10 imputed datasets were used.

#### Variable selection

Predictors for a multivariable regression model for heart rate were selected from the variables considered in the univariable models by a step-backward algorithm based on the Schwarz Bayesian Information criterion (BIC) for each imputed dataset. CCS class was used as a linear predictor in the multivariable models, following the observation of an almost linear increase in the univariable models. As a result 10 different models were obtained, one for each imputed dataset. Next, the following two-step variable selection procedure was used to obtain a set of significant variables. First, for each variable the number of times the variable appeared in the 10 models was calculated. Then, variables which appeared in at least half of the models (or in half the imputed datasets) were selected. The model containing selected variables was subsequently compared with smaller (nested) models based on the Wald test statistic calculated from the 10 imputed datasets. The corresponding p-values informed whether the contribution of a variable that had been omitted in the smaller model was statistically significant (p-value <0.05) or not.

## Results

### Patient characteristics

In the ProCor II database, 106 general practitioners recorded 1020 cases. Main patient characteristics of ProCor I [3] and ProCor II cohorts were very similar, with the exception of more cases of depression, less cases of chronic respiratory disease, and more beta-blocker treatments in ProCor II. Patient origin was from Vienna (26%), Lower Austria (25%), Upper Austria (13%), Styria (13%), Tyrol (8%), Carynthia (7%), Burgenland (4%), Salzburg (2%) and Vorarlberg (2%). ProCor I study data have been summarized [3]. Patient characteristics of the ProCor II cohort are shown in Table 1, with stratification by gender.

**Table 1:** Characteristics of the ProCor II study population by gender.

Characteristic	Observations (n = 1014)	Male (n = 680)	Female (n = 334)	p-value
Age (median (IQR))	1005	69 (60,76)	75 (68,81)	< 0.001
Physical activity (%)	972			< 0.001
Sedentary		99 (15.3)	83 (25.9)	
Light physical activity most weeks		244 (37.8)	128 (39.9)	
>20 min phys. act. up to three times weekly		227 (35.1)	94 (29.3)	
>20 min phys. act. >3 times weekly		76 (11.8)	16 (5)	
Smoking status (%)	954			< 0.001
current		149 (23.4)	40 (12.8)	
never		339 (53.3)	245 (78.3)	
past		148 (23.3)	28 (8.9)	
Depression = Yes(%)	939	133 (21.4)	128 (41)	< 0.001
Family history of CAD = Yes(%)	620	316 (76.1)	151 (75.5)	0.941
Previous stroke = Yes(%)	958	60 (9.4)	30 (9.5)	1.000
Type II Diabetes = Yes(%)	990	216 (32.6)	117 (36.2)	0.295
Hyperlipidemia = Yes(%)	978	563 (86)	249 (78.3)	0.003
Peripheral artery disease = Yes(%)	917	156 (25.4)	86 (28.8)	0.318
Systemic hypertension = Yes(%)	986	566 (85.9)	278 (86.1)	1.000
Chronic lung disease = Yes(%)	945	109 (17.3)	56 (18.1)	0.835

Percent (%) are in brackets; p-values were calculated using the chi-squared test for categorical variables (with continuity correction) and ANOVA for continuous variables.

As expected, female patients with stable ischemic heart disease were older than males (75 versus 69 years), less physically active (>20 min physical activity >3 times per week in 5.0% versus 11.8%), had less hyperlipidemia (78.3% versus 86.0%) and were less likely to be current or past smokers (21.7 versus 46.7%), had less previous coronary events/revascularizations (74 versus 84%) and were twice as likely to suffer from depression (41 versus 21.4%). 742 patients of ProCor I and II were diabetic, with a significantly higher heart rate than non-diabetic patients (heart rate 72.5±13.7 versus 69.0±12.0, p<0.001), on observation that has been associated with shorter survival in diabetics in the Euro Heart Survey [5]. They had also more angina and/or heart failure symptoms (45.4 versus 32.2% and 49.5 versus 38.2%), more atrial fibrillation (26.2 versus 19.3%) and higher heart rates (Table 2).

**Table 2:** Cardiovascular disease characteristics.

Characteristic	Obs.	Male	Female	p
Count	1014	680	334	
Any angina = Yes (%)	882	189 (32.2)	132 (45.4)	< 0.001
Angina episodes/week (median(IQR))	279	1.5 (1,3)	1.5 (1,2)	0.523
Nitroglycerine bottles/week (median(IQR))	279	1 (0,2.5)	1.5 (0,3)	0.266
Angina class	735			0.001
CCS0		241 (50)	84 (33.7)	
CCS1		131 (27.2)	90 (36.1)	
CCS2		73 (15.1)	51 (20.5)	
CCS3		26 (5.4)	19 (7.6)	
CCS4		11 (2.3)	5 (2)	
Atrial fibrillation = Yes(%)	992	128 (19.3)	85 (26.2)	0.016
Pacemaker = Yes(%)	997	42 (6.3)	25 (7.7)	0.500
CRT/Defibrillator = Yes(%)	977	26 (4)	10 (3.1)	0.647
Heart failure = Yes(%)	914	236 (38.2)	144 (49.5)	0.002
Heart failure symptoms	506			0.044
No heart failure		85 (26.8)	28 (15.1)	
NYHA I		83 (26.2)	56 (30.3)	
NYHA II		103 (32.5)	73 (39.5)	
NYHA III		37 (11.7)	24 (13)	
NYHA IV		9 (2.8)	4 (2.2)	
Heart rate median(IQR)	1001	70 (62,78)	72 (65,80)	0.018
Heart rate groups	1001			0.208
bpm > 70		317 (47.4)	172 (52.6)	
60 = < bpm < = 70		250 (37.4)	116 (35.5)	
bpm < 60		102 (15.2)	39 (11.9)	

Percent (%) are in brackets; p-values were calculated using the chi-squared test for categorical variables (with continuity correction) and ANOVA for continuous variables. Obs.—Observations, CCS—Canadian Cardiovascular Society Class of angina, CRT—cardiac resynchronisation, NYHA—New York Heart Association, IQR—interquartile range, bpm—beats per minute. The number of observations represents the sum of all yes and no answers. Percent indicate the relation of yes to no responses, for example 38% of males had heart failure, and 62% had no heart failure.

In accordance with these data, female patients were more likely to be on oral anticoagulants and anti-angina drugs, but less likely to be on lipid-lowering medications and/or dual platelet inhibitors (Table 3). Despite a higher rate of clinical heart failure symptoms and higher heart rates in female patients, there were no differences in the treatment with beta-blockers or with drugs exerting beta-blocking effects (Table 3). In addition, there were no differences in the use of beta-blockers or drugs exerting beta-blocking effects between primary and secondary care providers.

**Table 3:** Ischemic heart disease medications.

	Obs.	Male	Female	p
Count	1014	680	334	
Beta-blockers	1019			0.569
Atenolol		9 (1.3)	3 (0.9)	
Bisoprolol		256 (37.6)	134 (40.1)	
Metoprolol		79 (11.6)	38 (11.4)	
Nebivolol		112 (16.5)	47 (14.1)	
Carvedilol		66 (9.7)	31 (9.3)	
Others		12 (1.8)	12 (3.6)	
No beta-blocking agents		146 (21.5)	69 (20.7)	
Drugs with beta-blocking effect				
Amiodaron, Dronedaron, Sotalol	900	43 (7.1)	13 (4.5)	0.172
Ivabradin	954	27 (4.2)	15 (4.9)	0.781
Glycosides	970	31 (4.8)	25 (7.8)	0.078
ACE-Inhibitors/AT1-Blockers	972	513 (78.4)	241 (77)	0.672
Statins and lipid-lowering drugs	997	575 (86.3)	250 (76.7)	< 0.001
Calciumantagonists	1019			0.507
Diltiazem		12 (1.8)	11 (3.3)	
Verapamil		9 (1.3)	4 (1.2)	
Amlodipin		121 (17.8)	64 (19.2)	
Others		27 (4)	16 (4.8)	
None		511 (75.1)	239 (71.6)	
Anti-anginal Drugs				
Nicorandil	969	110 (16.8)	72 (23.2)	0.024
Long-acting nitrates	965	42 (6.5)	31 (9.7)	0.101
Ranolazin	961	6 (0.9)	6 (1.9)	0.338
Molsidomin	965	30 (4.6)	24 (7.7)	0.072
Oral anticoagulants	961	123 (19.2)	85 (27.1)	0.007
Platelet -inhibitors				
Aspirin	978	496 (76.4)	232 (71.6)	0.120
DAPT	954	173 (27.1)	60 (19.4)	0.012
Diuretics	845	282 (49.3)	148 (54.8)	0.156

Percent (%) are in brackets; p-values were calculated using the chi-squared test for categorical variables (with continuity correction) and ANOVA for continuous variables. Obs.—Observations, ACE—angiotensin converting enzyme, AT1—angiotensin 2 receptor type 1, DAPT—dual anti platelet therapy

### Heart rate control

An elevated heart rate is an established marker of cardiovascular risk [6–10]. Therefore, we analyzed heart rate control as a general read out for quality of care of stable ischemic heart disease in the Austrian stable CAD population depicted in ProCor I and ProCor II.

**Fig 1:**
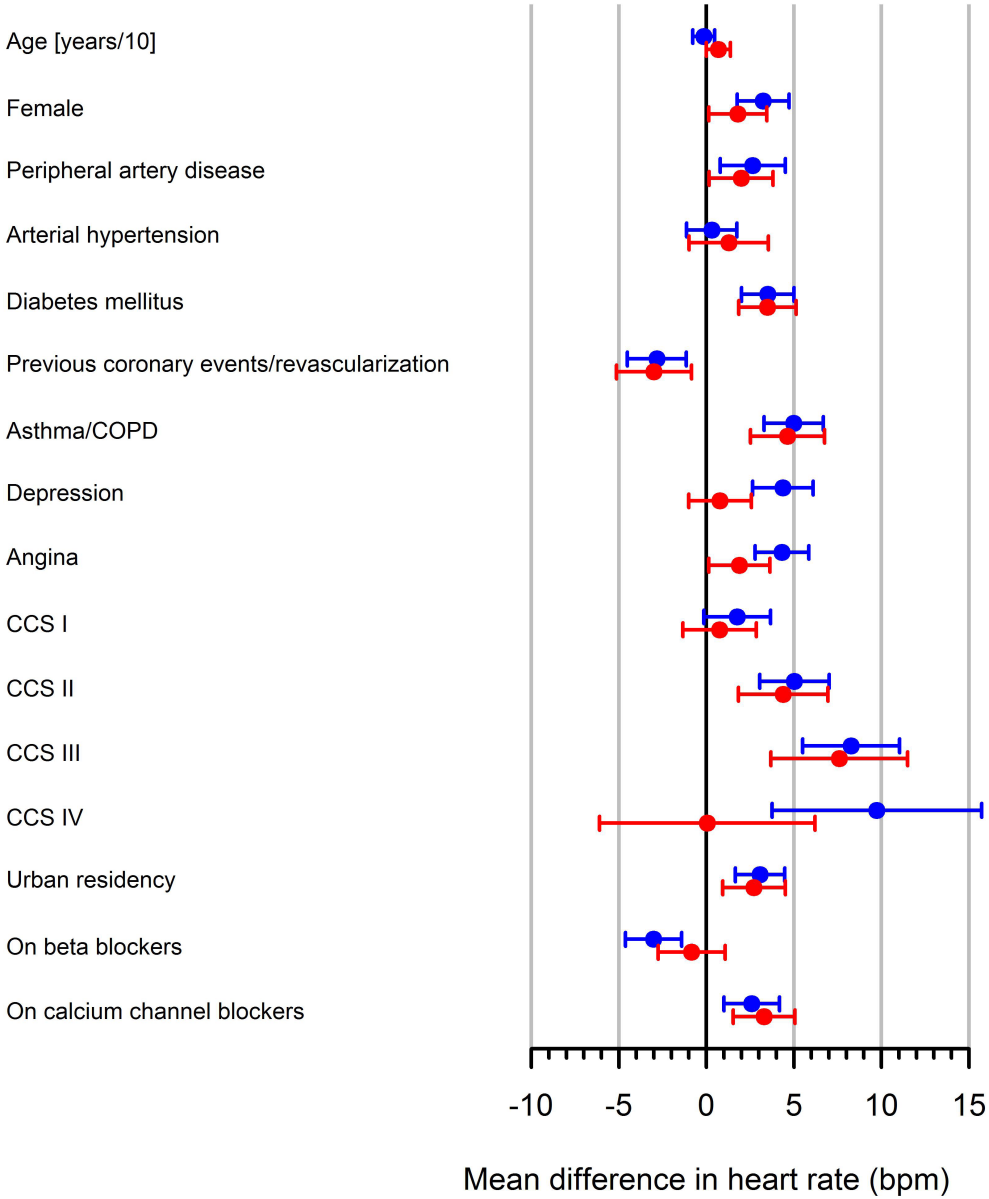
Regression estimates and confidence intervals of simple models analyzing predictors of heart rate in ProCor I (blue) and ProCor II (red). The effect of age is shown in HR change per 10 years. Female denotes female gender.

For 13 shared variables in ProCor I and ProCor II, individual-variable models were considered for data from ProCor I, data from ProCor II and pooled data from both studies. In this case, results from ProCor I and ProCor II showed good agreement in terms of the regression coefficient estimates and their confidence intervals (Fig 1).

**Fig 2:**
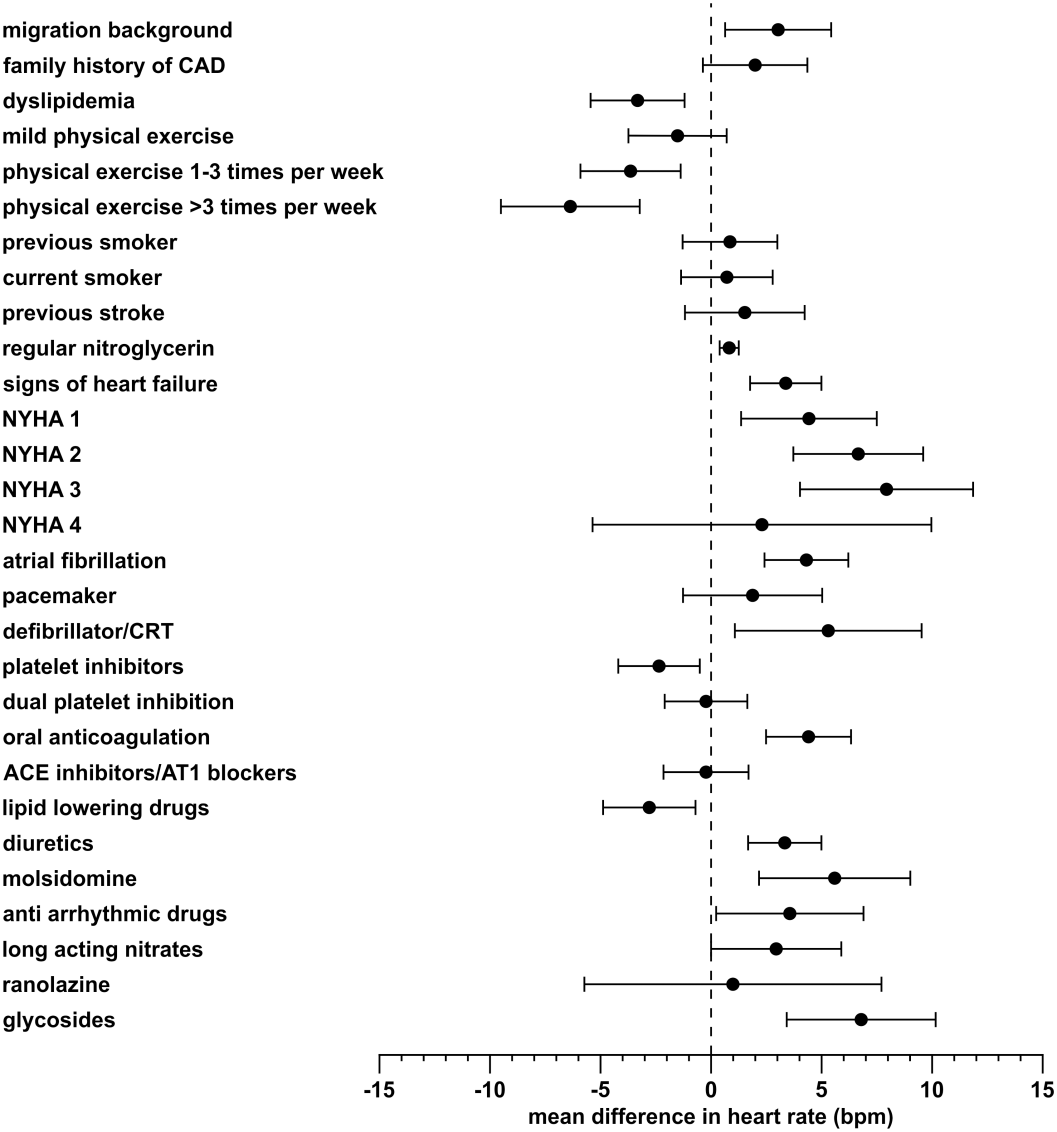
Regression estimates and confidence intervals of simple models analyzing predictors of heart rate using variables that were unique to the ProCor II dataset.

For the pooled data from both studies results were similar to those obtained from ProCor I alone, with a difference in significance for systemic hypertension and weekly angina incidence that were not significant in ProCor I, but significant in the pooled-data analysis. Among 23 variables that were unique for ProCor II 13 variables were significant at the 0.05 significance level in the simple regression model: migration background, dyslipidemia, intensive physical exercise, weekly use of nitroglycerine, heart failure, atrial fibrillation, defibrillator, aspirin, oral anticoagulation, diuretics, lipid lowering drugs and treatment with molsidomine (Fig 2).

**Fig 3:**
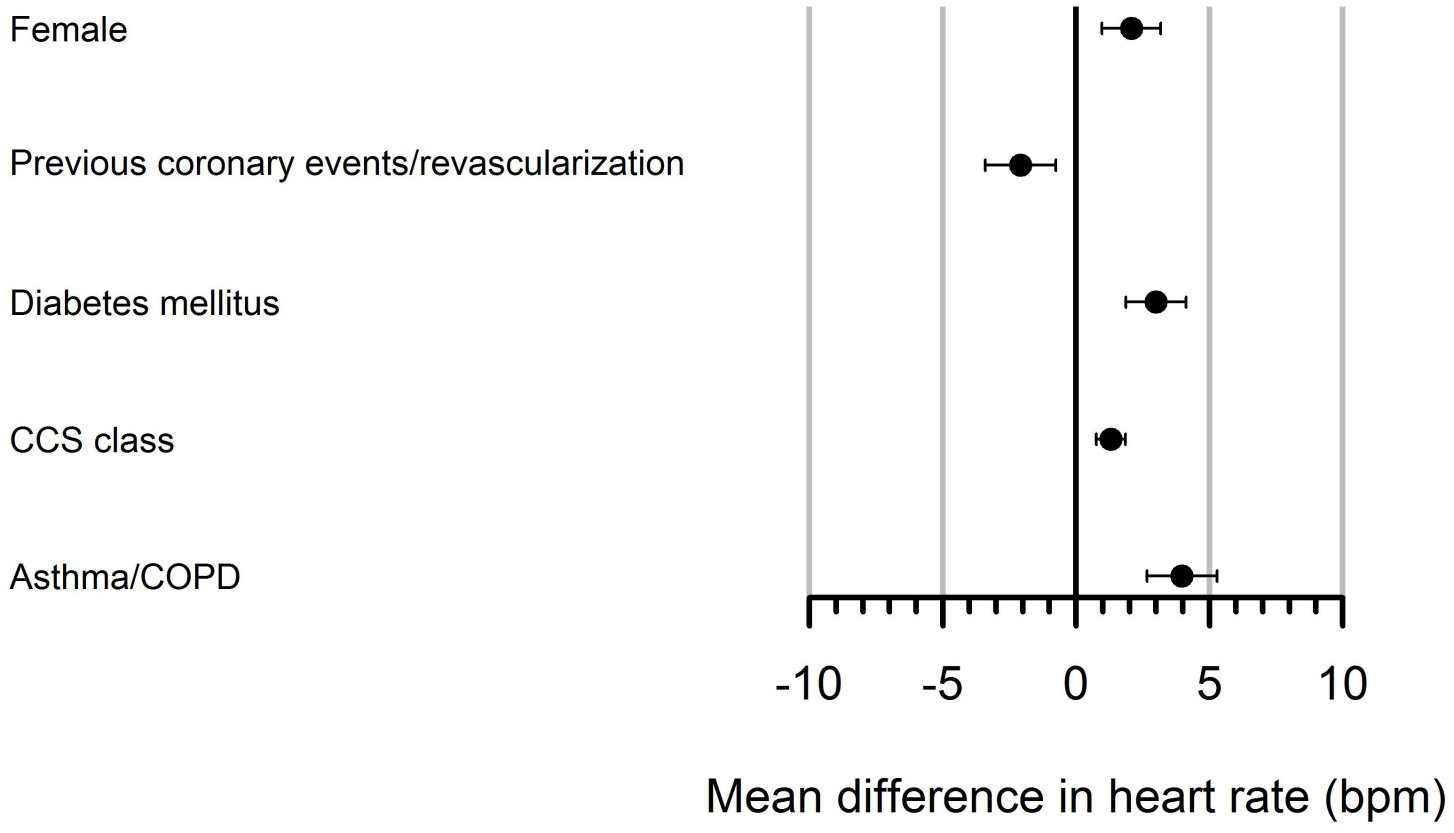
Regression estimates and confidence intervals resulting from multiple imputations and variable selections applied to the pooled data from ProCor I and ProCor II. Female denotes female gender.

Estimates (and 95% confidence intervals) for 13 common variables (age, gender, peripheral artery disease, systemic hypertension, type 2 diabetes mellitus, previous myocardial infarction or revascularization, COPD, depression, symptomatic angina in episodes per week or CCS class, beta blocker treatment, treatment with ivabradin and/or nicorandil, calcium channel blockers or long-acting nitrates) were assessed in simple models based on data from ProCor I, ProCor II, and pooled data from both studies. The analysis of the ProCor I dataset demonstrated urban residence, dyslipidemia and CCS class as predictors of elevated heart rate. The analysis of the ProCor II dataset using variable selection demonstrated urban residence, previous coronary events/revascularization, gender, and CCS class as predictors of elevated heart rate. The analysis of pooled data from ProCor I and ProCor II and a common set of variables from both studies demonstrated that in a multivariable model female gender, type 2 diabetes mellitus, higher CCS class and asthma/COPD were associated with elevated heart rate, while previous coronary events/revascularization were associated with lower heart rate (Fig 3).

## Discussion

In this unique cross sectional study of contemporary patients with stable chronic ischemic heart disease in Austria, a country with unrestricted access to healthcare, data on baseline characteristics of patients under ICD 10 diagnostic code I25.1 were collected by general practitioners (ProCor II). A previous database (ProCor I) that had been conducted with the support of 74 internal medicine specialists in 2009, had collected data on pre-existing and concomitant diseases, angina severity (CCS-class), frequency of angina and treatment. ProCor I was both compared and merged with ProCor II to yield a total patient number of 2300 patients. Main findings were that the clinical profile of female patients with chronic ischemic heart disease was at variance with that of male patients as they were older, had also more angina and/or heart failure symptoms and more depression. Because of the cross-sectional study design no outcome data were available, and therefore, heart rate was utilized as a surrogate of outcome. In the multivariable analysis of the pooled data, markers of disease severity and comorbidity such as CCS class, diabetes and asthma/COPD as well as gender remained predictors of a higher heart rate. Fig 2 illustrates that increases in resting heart rates were as high as 4.7 beats per minute in patients with asthma/COPD versus patients free of asthma/COPD, presumably due to use of beta-mimetic and or anti-cholinergic medications. For comparison, female gender and diabetes were associated with increased resting heart rates of 2 and 3 beats per minute, respectively. In addition to heart rates, women presumably derive increased risk from less-well treated hyperlipidemia, with 10.2% of untreated females compared with 5.8% untreated males (p<0.03).

While 74 internists had recorded 1280 patients in ProCor I, 106 out of 810 originally targeted general practitioners recorded 1020 cases in the ProCor II database. Although Austria has harmonized health care with no major regional differences in accessibility, care and cost, it is to be assumed that the patient populations derived from internal specialists including cardiologists differ from patients cared for by general practitioners. Furthermore, about 65% of the population are seen and managed by primary care physicians, and are referred to specialists only if needed [11]. In ProCor I urban residence was associated with higher heart rates. Baseline clinical characteristics including treatments were not different between ProCor I and ProCor II, and therefore, both datasets were pooled and common variables imputed in the analyses. Treatments in both studies were according to the guidelines with >90% of patients on beta-blockers, >75% of patients on ACE-Inhibitors or ARBs, >70% on aspirin, and about 80% on lipid-lowering medications.

In contrast to the large international Clarify registry, a large prospective longitudinal database capturing patients with stable ischemic heart disease [12], the percentage of female patients in our database (Tables 1–3) was higher (33% versus 22%), and women were older (75 years versus 66 years) which may be specific for the Austrian population. The profile of female patients with stable ischemic heart disease was otherwise well matched with the data from Clarify [12]. In this 330280-patient international database women were older than males (66.6 versus 63.4 years), more frequently diagnosed with diabetes (33% vs 28%), systemic hypertension (79% vs 69%), and had higher resting heart rates (69 vs 67 bpm), and were more sedentary. Smoking and a history of myocardial infarction were more common in men. Women were more likely to suffer from angina (28% vs 20%), but less likely to have undergone revascularization procedures.

We have also compared our data with the Reduction of Atherothrombosis for Continued Health (REACH) Registry. REACH enrolled consecutive outpatients age ≥45 years with established coronary artery disease, cerebrovascular disease, or peripheral artery disease, or with ≥3 atherothrombotic risk factors between December 2003 and June 2004. Therefore, REACH covered a wider population of patients with vascular disease than the current database, and was designed as an international prospective registry. However, a recent substudy of REACH focused on patients with stable coronary artery disease [13]. The data demonstrate that female gender was associated with more angina, and patients with angina had higher rates of future cardiovascular events compared with patients without angina [13].

In the Austrian female ischemic heart disease patients diabetes and systemic hypertension were not more common than in males, yet there was a generally higher prevalence of these two coronary risk factors in the Austrian population (>30% for diabetes and >80% for systemic hypertension, Table 1). Despite the higher burden of suffering in female patients, and the higher resting heart rate, female gender per se has not been found to increase cardiovascular risk [14].

The impact of resting heart rate on survival has been established by prior work [6, 15–17]. Given the available evidence from observational data ^6, 12^ and randomized trials ^7, 12^, it now appears appropriate to include reduction of elevated resting heart rate by lifestyle and pharmacological therapy as part of a secondary prevention strategy in patients with cardiovascular disease. In stable CAD, heart rate is a well-established determinant of ischemia. Elevations of heart rate during physical or emotional stress induce ischemia and angina [18]. However, while heart rate reduction with the *I*f inhibitor ivabradine improved prognosis in patients with chronic heart failure and left ventricular (LV) systolic dysfunction in the SHIFT study [19], ivabradine had no effect in patients with stable CAD without heart failure and LV systolic dysfunction in SIGNIFY. A major difficulty is to diagnose left ventricular diastolic dysfunction, particularly in mildly symptomatic patients with stable CAD. According to our data, only 15.1% of females (26.8% of males, Table 2) were free of heart failure symptoms, illustrating the overall high percentage of clinical heart failure in a stable CAD population. One might speculate that in any condition of chronic stable CAD there is a degree of left ventricular dysfunction, diastolic or systolic. More sensitive imaging techniques in the future will demonstrate the degrees of left ventricular functional compromise in CAD and their effect on resting heart rate.

Limitations of our study were the cross-sectional design and the lack of data on left ventricular function and renal function. It is unclear why a previous coronary event/revascularization was a predictor of a lower heart rate. One explanation may be that those patients were receiving more attention and more intense treatments with heart rate-modifying drugs. Precise information on medication adherence was not available in this study, and no other direct oral anticoagulants than dabigatran were observed. On the other hand, the strength of our study is that it provides important information about patients with stable CAD in Austria, avoiding referral bias by using two different samples, one derived from internal medical specialists, and one derived from primary care physicians.

Taken together, this unique Austrian study on chronic stable CAD is providing insights into characteristics, treatments and heart rate control of unselected patients with stable ischemic heart disease.

## Supporting information

S1 File ProCorII_Datensatz_3Feb2014.csv contains the data underlying this work and is provided as supplementary information. (XLSX)
